# Editorial: Chinese Only Children: Advantaged or Disadvantaged?

**DOI:** 10.3389/fpsyg.2021.742186

**Published:** 2021-08-23

**Authors:** Shengjie Lin, Bin-Bin Chen, Toni Falbo, Carlton J. Fong, Jiajun Guo

**Affiliations:** ^1^Center for Emotional Intelligence, Yale University, New Haven, CT, United States; ^2^Department of Psychology, Fudan University, Shanghai, China; ^3^Department of Educational Psychology, University of Texas at Austin, Austin, TX, United States; ^4^Department of Curriculum and Instruction, Texas State University, San Marcos, TX, United States; ^5^School of Psychology and Cognitive Science, East China Normal University, Shanghai, China

**Keywords:** Chinese only children, parent-child relationships, family resources, birth order, sibship size

Only children are individuals who have grown up without siblings. China now has the highest number and percentage of only children in the world due to their policy that strongly encouraged young families to have solely one child. This policy was known as the One-Child Policy (OCP), and soon after it was launched in 1979, popular media aroused concern about only children, stereotyping them as “Little Emperors.” Chinese parents and grandparents were assumed to overindulge only children, leading to undesirable outcomes, such as failure to live up to family obligations. The articles in this Research Topic address topics of potential concern about Chinese only children and their families, focusing on their relationships with their parents.

In “Who Benefits From Being an Only Child? A Study of Parent–Child Relationship among Chinese Junior High School Students,” Liu and Jiang explored the family relationships of only children, compared with children with siblings. In addition, they took another step forward by examining the birth-order effects and the gender-composition effects. They found that only children had a significant advantage in developing positive parent-child relationships when compared to non-only children (including those from two-child families). However, the only-child advantage disappeared after comparing only children to last borns of multiple-child families. In addition, the only-child advantage was gender-specific in Chinese families: compared to boys, girls benefited more from being only children. At last, the study further examined the effects of gender-composition and found that having older sisters was more advantageous than being an only child—this was true only for boys; it also found that having younger siblings was more disadvantageous than being an only child—this was especially true for girls. The findings implied that despite daughters' status has been improved in one-child families in China, son preference and daughter discrimination still persist in Chinese multiple-child families.

Continuing the discussion of the parental relationships of Chinese only children, Chen et al. explored another dimension of these relational dynamics in their article, “Adaptations to the One-Child Policy: Chinese Young Adults' Attitudes Toward Elder Care and Living Arrangement After Marriage.” Although many studies tend to focus on how parents influence the psychological well-being and behaviors of their only children, this study sheds light on the other direction of this seemingly reciprocal relationship. In a large sample of Chinese young adults from various cities in China, Chen and colleagues examined the attitudes, worries, and co-residing behaviors of only children and children with siblings. Interestingly, their sample endorsed low levels of worrying about their parents' elder care overall with no meaningful differences in their attitudes toward parental care between adults with and without siblings. However, among married young adults, only children were more likely to co-reside with parents than their counterparts with siblings. Reasons for only children to co-reside with parents were not based on concerns for parents' elder care but rather focused on receiving help from their parents in areas such as childcare (caring for grandchildren). Findings from this study provides additional nuance in our understanding of Chinese only children and the role of educational level in their complex mindsets toward aging parents and multigenerational concerns.

The article by Qian et al. “Assessing Mothers' Parenting Stress: Differences between One-and Two-child Families in China,” extended the Research Topic further by examining degrees of mother's stress, comparing mothers of one and two children. All the children ranged in age from 3 to 7 years and parenting stress was measured by a revised version of the Parenting Stress Index-Short Form. Mother's parenting stress was found to be higher in two-child families than one-child families; however, stress was also found to be significantly higher for parents of firstborns than parents of later-born children, and when there was a shorter age gap between children. Income was found to be associated with stress, with mothers of less income experiencing more stress, but no effects for mothers' educational level was found on parenting stress.

Past research has established that only daughters benefit from China's one-child policy due to the lack of competition between them and their potential male siblings. But few studies have looked beyond the first-generation to examine whether this advantage would expand to only daughters' own families when they grow up, get married, and have their own children. Exploiting a large dataset containing a sample of 1,007 fathers and 2,168 mothers born between 1975 and 1985, Wang and Feng explored the empowerment of married only daughters in their article, “Family Resource Dilution in Expanded Families and the Empowerment of Married Only Daughters: Evidence from the Educational Investment in Children in Urban China.” Wang and Feng examined an important index reflecting how families distribute resources among siblings—educational investment, which could also be used to test the resource dilution theory. Wang and Feng's findings revealed that Chinese families still tend to sacrifice the interests of married daughters to ensure support for their adult sons. However, it also illustrates that married only daughters could still connect to their parents' resources, giving them a relatively dominant position for decision-making regarding the family's educational expenditure on their own children. These findings call for more research on the differences between only and non-only children's family dynamics and related mechanisms.

## Future Directions

The Chinese government officially ended the OCP in 2016, motivated largely by concern about future labor shortages and the potential for a substantial population decline by 2050. The Chinese government replaced the OCP with a Two-Child Policy in 2016 and most recently, a Three-Child Policy in 2021. Future researchers should extend their focus to sibling effects on the development of children from diverse backgrounds in China, such as the children from the many nationalities within China and rural children left behind by parents who work in cities. Additionally, the fertility intentions and behaviors of young couples who are both only children in the era of Two/Three Child Policies requires more exploration. There is ample room for future discoveries about the advantages and disadvantages of growing up without siblings.

## Author Contributions

All authors listed have made a substantial, direct and intellectual contribution to the work, and approved it for publication.

## Conflict of Interest

The authors declare that the research was conducted in the absence of any commercial or financial relationships that could be construed as a potential conflict of interest.

## Publisher's Note

All claims expressed in this article are solely those of the authors and do not necessarily represent those of their affiliated organizations, or those of the publisher, the editors and the reviewers. Any product that may be evaluated in this article, or claim that may be made by its manufacturer, is not guaranteed or endorsed by the publisher.

